# Exploration of treatment after multiline chemotherapy in recurrent head and neck cancer: Case report

**DOI:** 10.1097/MD.0000000000047059

**Published:** 2026-01-16

**Authors:** Yang Wang, Chaonan Huangfu, Jiaxing Jiang, Wei Dai

**Affiliations:** a Oncology Department of Nanjing BenQ Hospital, Nanjing, Jiangsu, China.

**Keywords:** chemotherapy, head and neck cancer, multi-line, polymeric micellar paclitaxel, recurrence

## Abstract

**Rationale:**

Treatment of recurrent/metastatic head and neck squamous cell carcinoma (R/M HNSCC) remains challenging, especially after resistance develops to conventional chemotherapy, radiotherapy, and targeted agents. Novel formulations, such as polymeric micellar paclitaxel (pm-Pac), which offer a Cremophor-free delivery system, warrant investigation in salvage settings to overcome resistance and improve outcomes.

**Patient concerns:**

Two patients with locally advanced HNSCC experienced multiple disease recurrences following several surgical resections. Both had received prior taxane-based chemotherapy alongside radiotherapy, anti-EGFR therapy, and immunotherapy, yet developed progressive disease, highlighting the clinical dilemma of managing multiply relapsed, treatment-refractory HNSCC.

**Diagnoses:**

Both patients were diagnosed with R/M HNSCC, demonstrated low PD-L1 expression (TPS 2% and 3%, respectively), and negative EBER status.

**Interventions:**

Following progression on multiple prior lines of therapy, both patients received salvage regimens containing pm-Pac in combination with platinum, fluorouracil, and either an immune checkpoint inhibitor (tislelizumab, case 1) or a tyrosine kinase inhibitor (lenvatinib, case 2). The treatment was administered for 6 cycles, followed by maintenance therapy.

**Outcomes:**

The pm-Pac-based combination therapy achieved significant tumor regression. In case 1 (ear canal primary), the target lesion was reduced by 43.1%, resulting in a progression-free survival (PFS) of 8.2 months. In case 2 (laryngeal primary), a 59.1% reduction was observed, with a PFS of approximately 11 months. Treatment was generally tolerable, with manageable grade 2 neutropenia as the primary adverse event.

**Lessons:**

These 2 cases suggest that reintroducing paclitaxel in its novel polymeric micellar formulation, pm-Pac, within a combination regimen may overcome prior resistance and provide meaningful disease control in heavily pretreated R/M HNSCC patients. This approach represents a potentially viable salvage strategy, warranting further clinical investigation to confirm its efficacy and optimal integration into the treatment sequence for refractory HNSCC.

## 1. Introduction

Head and neck cancers represent a heterogeneous group of malignancies affecting the upper aerodigestive tract, salivary glands, and thyroid glands. Head and neck squamous cell carcinomas (HNSCCs) constitute approximately 90% of cases and primarily originate from the mucosal epithelium of the oral cavity, oropharynx, hypopharynx, and larynx.^[1]^ A subset of HNSCCs is associated with human papilloma virus infection, whereas more aggressive human papilloma virus-negative variants are strongly linked to tobacco use.^[2]^ Most patients with HNSCC are diagnosed at advanced stages and often present with functional impairments or cervical masses. More than 70% of patients with HNSCC exhibit locally advanced disease at initial diagnosis,^[3]^ leading to poor survival outcomes owing to limited therapeutic options or treatment failure.^[4]^

Multimodal therapies are essential in patients with locally advanced HNSCC. Traditional chemotherapeutic agents, such as cisplatin, 5-fluorouracil, paclitaxel, and docetaxel, are frequently used for induction therapy or in combination with definitive radiotherapy.^[5]^ However, development of resistance to chemotherapy and radiotherapy remains a significant challenge. Overexpression of the epidermal growth factor receptor is observed in up to 90% of HNSCCs, with higher levels correlating with reduced survival rate. For locally advanced HNSCC, the combination of cetuximab with radiotherapy has demonstrated superior disease control rates compared to radiotherapy alone (24.4 months vs 14.9 months, *P* = .005).^[6]^ In recurrent or metastatic (R/M) HNSCC, the EXTREME regimen improves progression-free survival (PFS) and overall survival (OS).^[7]^ Over the past decade, immunotherapy has advanced rapidly, particularly for solid tumors, such as melanoma, lung cancer, and esophageal and gastric cancers. The Keynote-048 trial marked a pivotal shift in the treatment landscape of R/M HNSCC.^[8]^ Based on its favorable outcomes, the Chinese Society of Clinical Oncology guidelines have approved pembrolizumab in combination with chemotherapy for the treatment of R/M HNSCC and pembrolizumab monotherapy for patients with CPS ≥ 1. This report presents 2 cases of HNSCC with multiple recurrences following surgery, highlighting successful disease control through combined sequential polymeric micellar paclitaxel (pm-Pac) treatment. pm-Pac is a novel Cremophor EL-free polymeric micelle formulation of paclitaxel that was originally introduced in Korea for the treatment of metastatic breast cancer and non-small cell lung cancer (NSCLC).^[9,10]^ In this special structure, a hydrophobic drug is loaded in the hydrophobic core of micelles, which has 2 benefits: avoiding the use of toxic solubilizing agents and improving the solubility of poorly soluble anti-tumor compounds.^[11,12]^ In our study, both patients underwent multiple lines of chemotherapy and had received prior paclitaxel treatment. Nonetheless, subsequent treatment with pm-Pac combination therapy managed to control the tumor, which also provides some references for subsequent treatment in similar situations.

In this study, tumor response after treatment was evaluated according to RECIST version 1.1,^[13]^ and tumor measurements for each patient were performed manually on MRI images. The sum of the longest diameters of the target lesions of each patient was recorded, and PFS and objective response were evaluated.

### 1.1. Ethical compliance

All procedures performed in this study involving human participants were in accordance with the ethical standards of the institutional and/or national research committee and with the 1964 Helsinki Declaration and its later amendments or comparable ethical standards.

## 2. Case report

### 2.1. Case 1

**Figure 1.**
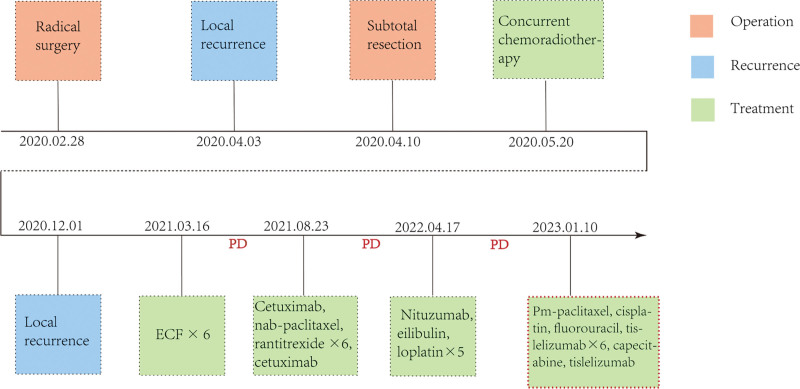
Timeline of the patient management for case 1. ECF, chemotherapy regimen of doxorubicin hydrochloride liposome, cyclophosphamide, fluorouracil. PD = progressive disease.

**Figure 2.**
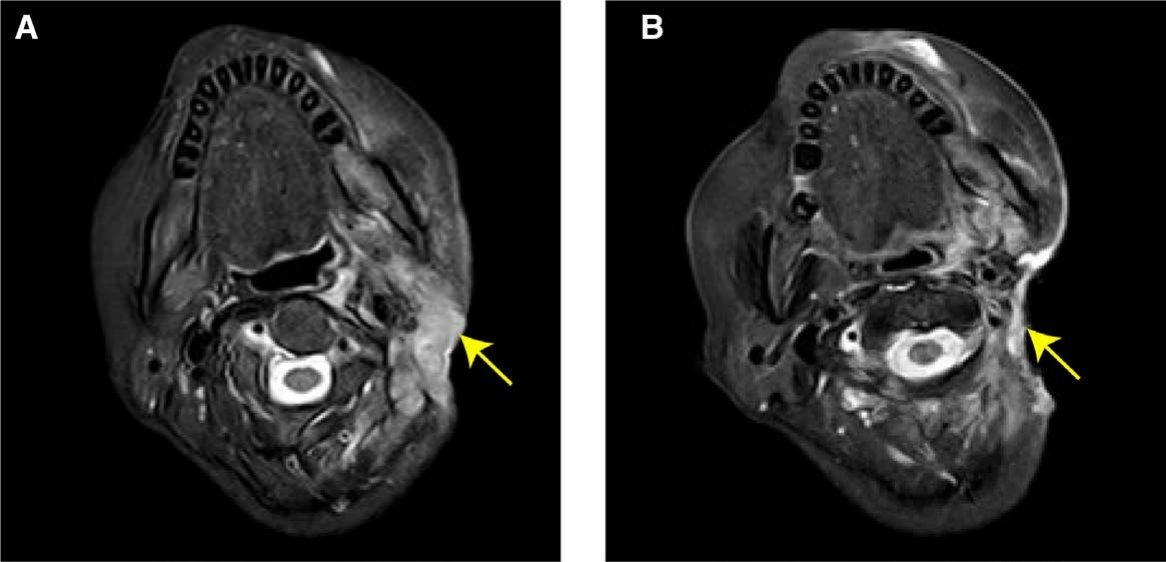
MRI scan of case 1 before and after use of pm-Pac combined therapy. The lesion was in the postoperative area around left middle ear, with the inner edge reaching the left side of the nasopharynx and the outer edge reaching the left outer auricle. It shows uneven enhancement in T1 enhanced signal. (A) The lesion progressed into 5.8 cm × 2.9 cm, time: 2023-01-07; (B) after 6 cycles of pm-Pac combined therapy, the lesion reduced to 3.3 cm × 1.0 cm, time: 2023-07-27. MRI = magnetic resonance imaging.

**Figure 3.**
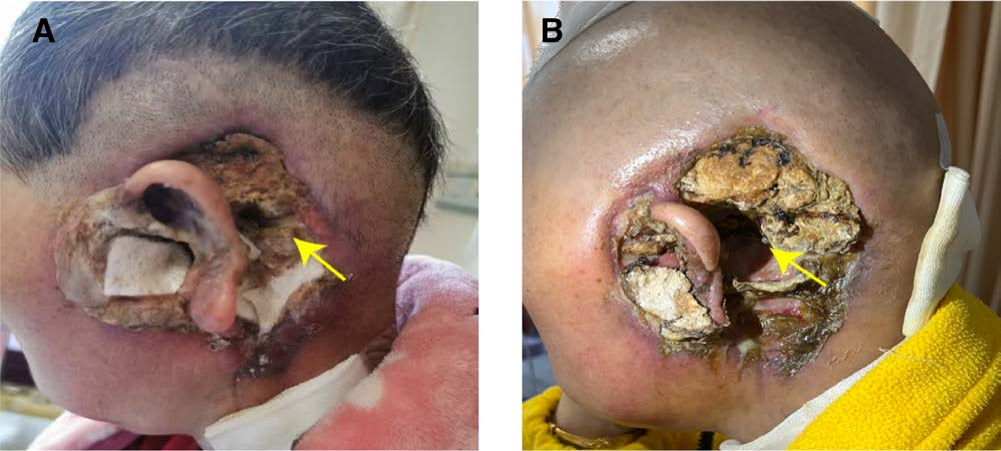
The lesion of case 1 was diffused around the left ear. (A) The lesion evaded the ear with much exudation. (B) The lesion behind the ear had diminished, the exudation was reduced.

A 52-year-old female patient initially presented with symptoms of hearing loss, left facial paralysis, and purulent discharge with bleeding behind the left ear in 2020. Radical surgical procedures including left ear mastoidectomy, modified radical mastoidectomy, and left external auditory canal plasty were performed. Postoperative pathology confirmed a moderately well-differentiated squamous cell carcinoma in the left ear mastoid region, with Ki-67 (60%+) and EBER (−). Two months later, MR revealed a residual mass (43 mm × 29 mm × 33 mm) involving the left tentorium, posterior margin of the parotid gland, and lower margin of the auricle, with extension around the petrous segment of the left internal carotid artery. Subsequently, subtotal resection was performed. Following the subtotal resection, the patient underwent concurrent radiotherapy and chemotherapy. Eight months after the second operation, MRI revealed tumor recurrence at the surgical site, accompanied by skin dehiscence with pus behind the left ear. Biopsy confirmed recurrence, and immunohistochemistry revealed positive progressive disease (PD)-L1 protein expression (TPS 2%). The patient received 6 cycles of “doxorubicin hydrochloride liposomes, cyclophosphamide, and fluorouracil” chemotherapy. Due to local disease progression, treatment was switched to “cetuximab + albumin-bound paclitaxel + raltitrexed” for 6 cycles, followed by maintenance therapy with cetuximab. After 4 cycles of maintenance, disease progression was observed, prompting a change to “necitumumab + eribulin + lobaplatin” for 5 cycles. On January 8, 2023, due to tumor progression, treatment was further adjusted to “pm-Pac + cisplatin + fluorouracil + tislelizumab” for 6 cycles (all drugs were administered intravenously for each 21-day cycle, pm-Pac 230 mg/m^2^, d1; cisplatin 60 mg/m^2^, d1; fluorouracil 600 mg, d1–5; tislelizumab 200 mg), followed by maintenance therapy with capecitabine (1000 mg/m^2^, po, d1–14) and tislelizumab (200 mg d1) each 21-day cycle (Fig. 1). After the first cycle of treatment, the patient developed grade 2 neutropenia, which resolved without intervention, and we did not modify the dose of any drug in the subsequent treatment. The target lesion was reduced by 43.1% after 6 cycles of treatment compared to the baseline measurement (Figs. 2 and 3). No serious drug-related adverse reactions were observed during the treatment. The lesion was control for 8.2 months. She stopped the maintenance treatment due to fatigue in October 2023, the lesion gradually increased in size, and the patient could not tolerate continued anti-tumor therapy and eventually died in August 2024 due to mass rupture and bleeding.

### 2.2. Case 2

**Figure 4.**
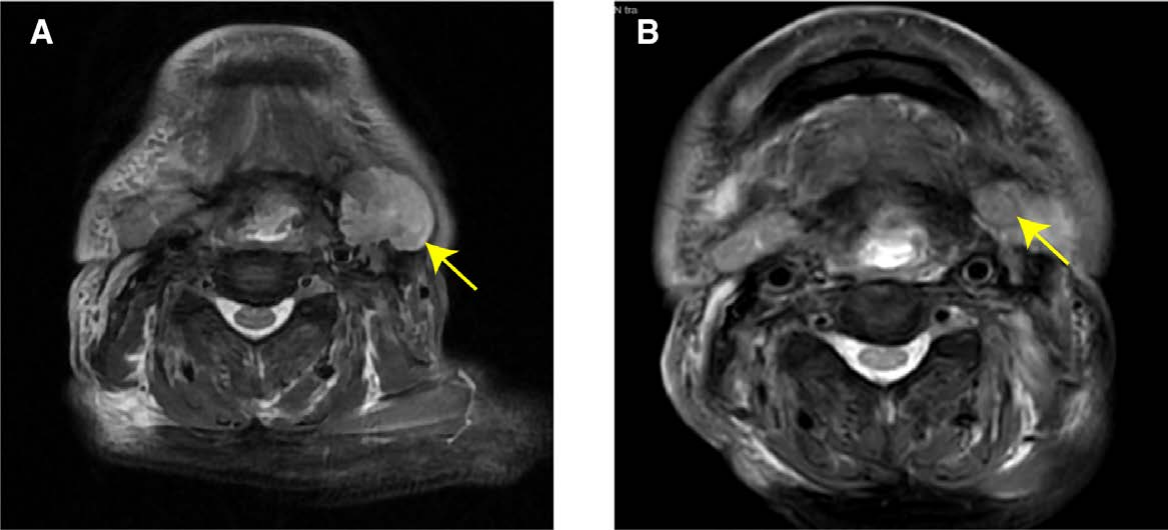
MRI scan of case 2 before and after use of pm-Pac. The lesion was next to the vascular sheath in the left neck, it showed uneven high signal of STIR sequence. (A) The lesion progressed into 3.7 cm × 4.4 cm, time: 2023-03-10; (B) after 6 cycles of pm-Pac combination therapy, the lesion reduced to 1.5 cm × 1.8 cm, time: 2023-12-11. MRI = magnetic resonance imaging, pm-Pac = polymeric micellar paclitaxel, STIR = short tau inversion recovery.

**Figure 5.**
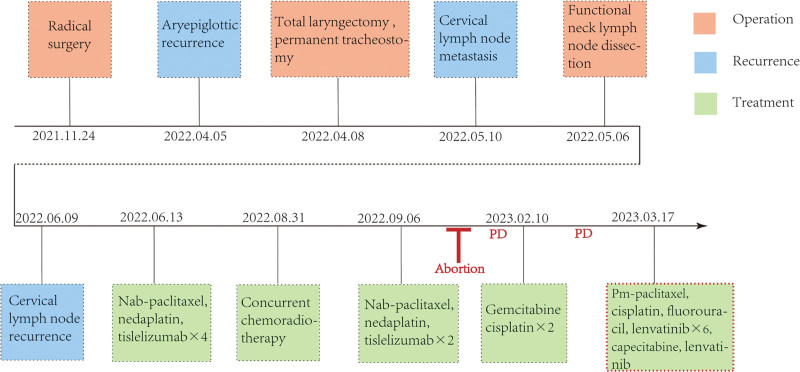
Timeline of the patient management for case 2. PD = progressive disease.

A 67-year-old male patient presented in September 2021 with hoarseness and laryngoscopic findings of neoplastic growth in the right vocal cord. Laryngeal cancer was diagnosed on the basis of laryngoscopy and CT imaging. The patient underwent “vertical hemilaryngectomy + bilateral neck lymph node dissection + tracheotomy.” Pathological examination confirmed squamous cell carcinoma with Ki-67 expression (hot spot 80%+) and EBER (−). Immunohistochemical analysis revealed PD-L1 expression (TPS 3%). Tumor recurrence occurred 7 months post-surgery, prompting a second total laryngectomy and permanent tracheostomy. One month later, the patient presented with a neck mass that was accompanied by dysphagia. CT tomography revealed multiple annularly enhanced enlarged lymph nodes bilaterally in the cervical region. A functional neck lymph node dissection was performed. Postoperative pathology indicated tumor invasion of salivary gland tissue and 1 metastatic lymph node (1/7). One month after the third resection, PET-CT revealed multiple enlarged lymph nodes in the right parotid gland area, with some lesions infiltrating the surrounding soft tissue, confirming tumor recurrence. The patient received 4 cycles of “albumin-bound paclitaxel + nedaplatin + tislelizumab,” followed by concurrent radiotherapy and chemotherapy. Owing to PD, treatment was switched to “gemcitabine + cisplatin” for 1 cycle; however, it was discontinued because of intolerance to side effects. The patient experienced significant pain in the left side of the face and head in March 2023. Third-line therapy with “pm-Pac + cisplatin + fluorouracil” combined with lenvatinib was initiated, with each 21-day cycle (pm-Pac 230 mg/m^2^, d1; cisplatin 60 mg/m^2^, d1; fluorouracil 600 mg, d1–5; lenvatinib 8 mg po qd). The target lesion size was reduced by 59.1% after 6 cycles of treatment (Fig. 4). Subsequently, maintenance therapy with capecitabine (1000 mg/m^2^, po, d1–14) and lenvatinib (8 mg, po, qd) was administered (Fig. 5). During pm-Pac concurrent treatment, the patient developed grade 2 neutropenia. Following administration of granulocyte colony-stimulating factor, the patient recovered and continued the original dosage until the completion of 6 cycles of treatment. The patient achieved PFS of approximately 11 months. In February 2024, the patient developed axillary lymph node enlargement although the mass in the left neck remained stable. The patient died in December 2024 due to axillary lymph node metastasis, ulceration, and infection.

## 3. Discussion

The 5-year survival rate of locally advanced HNSCCs remains below 50%,^[4]^ ranging from 49% for laryngeal cancer to 25% for hypopharyngeal cancer.^[14]^ In 2016, the US Food and Drug Administration approved the first anti-PD-1 agent, nivolumab, for the treatment of platinum-refractory recurrent and/or metastatic HNSCC.^[15]^ Subsequently, the Food and Drug Administration approved pembrolizumab as a first-line treatment for HNSCC due to the Keynote 048 clinical trial, which proved that pembrolizumab with chemotherapy improved OS compared to cetuximab with chemotherapy in the total population, especially for CPS ≥ 20 population.^[8]^

The proportion of patients exhibiting high PD-L1 expression (defined as CPS ≥ 20 and TPS ≥ 50%) ranged from 30% to 43%.^[8,16]^ However, therapeutic gaps still need to be addressed for patients with low or negative PD-L1 expression and those refractory to PD-L1 inhibitors.

Chemotherapy remains the cornerstone of treatment for advanced-stage HNSCC. However, the development of chemoresistance poses a significant clinical challenge. Various mechanisms underlying chemoresistance of HNSCC have been investigated.^[17]^ Taxanes, including paclitaxel and docetaxel, are widely recommended in clinical guidelines. Resistance to conventional taxanes has driven research into novel formulations aimed at improving efficacy, tissue penetration, and reducing toxicity.

This study describes the treatment experiences of 2 patients with advanced HNSCC. The first case involved squamous cell carcinoma of the left ear canal, a rare primary malignancy in <0.2% of all head and neck cancers.^[18]^ Despite undergoing 2 surgical procedures, the tumor recurred postoperatively and infiltrated the adjacent soft tissue and bone, precluding radical excision. Following multiple lines of chemotherapy and anti-epidermal growth factor receptor targeted therapy, pm-Pac, a novel taxane formulation, was reintroduced after a 1-year interval following albumin-bound paclitaxel treatment. Encouragingly, the lesions demonstrated sustained alleviation, with disease control maintained for 8.2 months when combined with capecitabine and immune checkpoint inhibitors. In China, pembrolizumab combined with chemotherapy was approved as the first-line treatment for R/M HNSCC in December 2020, when the PD-L1 CPS was ≥20. Therefore, we did not choose ICIs as the first-line therapy because of PD-L1 expression (TPS 2%). In the later line of treatment, there is no established standard treatment, and we tried pm-Pac along with our native anti-PD-1 antibody, tislelizumab, which has demonstrated promising efficacy in some studies for palliative anti-tumor treatment. The second case involved a patient with laryngeal cancer who underwent 3 operations following recurrence. Despite previous treatment with albumin-bound paclitaxel, disease control was achieved following reinitiation of paclitaxel micelles after a 4-month interval. Both patients in this study had locally advanced HNSCCs and underwent comprehensive anticancer therapy including chemotherapy, radiotherapy, targeted therapy, and immunotherapy. However, challenges related to drug resistance persist, which underscores the need for innovative treatment strategies.

In a study evaluating first-line treatment for NSCLC, patients receiving pm-Pac in combination with cisplatin demonstrated significantly improved outcomes compared to those treated with nab-paclitaxel plus cisplatin (overall response rate: 50% vs 26%; *P* < .0001). The most common treatment-related adverse effect of pm-Pac is myelosuppression, particularly neutropenia and thrombocytopenia, which is not significantly different from that of albumin-paclitaxel. The digestive system reactions include nausea, vomiting, and poor appetite, which are lower than those of albumin-paclitaxel (9% vs 18%).^[19]^ Consequently, pm-Pac has been approved by the Chinese Society of Clinical Oncology as a first-line treatment for NSCLC. Both the patients in this study developed neutropenia after treatment with pm-Pac. However, they were all within the controllable range (grade 2) and did not affect continued treatment.

The 2 patients with positive PD-L1 expression received tislelizumab during distinct treatment phases. In China, approximately 50% of HNSCCs cases exhibit high PD-L1 expression.^[16]^ Tislelizumab combined with chemotherapy has shown promising efficacy in this setting.^[20–22]^ Notably, neither of these patients belonged to the high PD-L1 expression subgroup; thus, tislelizumab was administered during specific treatment stages, and the regimen was adjusted upon disease progression due to drug resistance.

Despite advancements in therapeutic strategies, unmet needs persist in the management of recurrent or metastatic HNSCC. In this report, we illustrated the potential of a novel formulation that exhibits favorable efficacy and acceptable tolerability in patients with advanced HNSCC. Although these 2 cases experienced extended OS after multiple lines of treatment, they were only individual cases, the treatment regimen involved other drugs (pm-Pac was combined with cisplatin, 5-FU, ICI, and/or lenvatinib), and their differences in previous treatments. Polymeric micelles have emerged as a highly promising delivery system for therapeutic compounds such as drugs, imaging agents, genes, and combination agents,^[23–25]^ and we look forward to more high-level evidence to confirm the efficacy of the polymeric micelle delivery system not only in cancer treatment but also in other disease settings.

## Author contributions

**Conceptualization:** Yang Wan.

**Methodology:** Chaonan Huangfu.

**Validation:** Jiaxing Jiang.

**Visualization:** Jiaxing Jiang.

**Writing – original draft:** Chaonan Huangfu.

**Writing – review & editing:** Yang Wang, Wei Dai.
